# Abstracts from German Journals

**Published:** 1873-02

**Authors:** Ch. Rauschenberg

**Affiliations:** Atlanta, Ga.


### ABSTRACTS FROM GERMAN JOURNALS.

BY CH. RAUSCHENBERG, M.D., ATLANTA, GA.

### *ON THE OPERATIVE TREATMENT OF PLEURITIC EXUDATIONS.*

Dr. Ludwig Lichtheim, Assistant at the Surgical Klinik at Halle, is the author of a valuable article on this subject, the most important points of which are contained in the following abstract:

Dr. Lichtheim first calls attention to the fact that the improved pathological conceptions of the last ten years, with their correcting and remodeling influences on therapeutical principles generally, have also brought about a material change in the therapeutical efforts of physicians in the treatment of local morbid conditions occurring in the course of internal diseases, and that the old faith in the efficiency of general treatment for the removal of such conditions is fast giving away to an improved system of local therapeutics, in which operative surgery is destined to occupy an important position.

This tendency of the age has effected the operation of thoracentesis and its therapeutical relation to the treatment of pleuritic exudations very essentially, and has demonstrated that it must henceforth be looked upon not as a last resort, or a mere palliative measure, but as a surgical remedy of very great and very general curative usefulness in the treatment of this morbid condition.

Heretofore this operation has almost exclusively occupied the position of a mere palliative measure. Excessive danger of suffocation, caused by the compression of the lung on the diseased side and impeded expansion of the other by the misplaced heart and mediastinum, on the one hand, and the danger threatened from the burrowing of pus when bulging of the intercostal integuments indicated pleural abscess on the other, have so far been, in a majority of instances, the phenomena under which thoracentesis has been performed, as they were the only generally-accepted indications under which this operation was considered applicable.

As a curative measure, aiming directly at the improvement of the morbid condition *per se*, this operation—although simple, safe, painless, and often life-saving—was first practiced and recommended in France by the pupils of the great Trousseau, one of its earliest and most zealous advocates in this sense; but it has only acquired the general acceptation of the medical world in the last few years. The labors of Kussmaul and Bartels, in Germany, have, however, the exclusive merit of having first clearly demonstrated the different relations which this operation as a remedy bears to the different pathological sequels of pleuritis, and have established the leading and now generally accepted principles which govern its use and execution.

The proper use of thoracentesis for the treatment of pleuritic exudations is based upon a correct understanding of the differences in the treatment required by purulent effusions, empyemata, on the one side, and that of non-purulent, mere serous effusions, on the other.

Resorption of the effused serum frequently takes place; but complete resorption of pus, constituting an empyema of any recognizable size, is a very doubtful occurrence, which has at least never been established by actual observation; while it is a well-known pathological fact that purulent deposits remain within the pleural cavity often for very long periods of time, either as fluid pus or a more or less inspissated substance, subject to or engaged in a process of cheesy degeneration, according to the degree of separation of the fluid parts of the pus from its solid morphological elements by resorption of the former.

The possibility of a perforation of the thorax or the pulmonal pleura and lungs, by which the empyema might be emptied and the disease spontaneously cured, can not be taken into consideration when the question of the necessity of thoracentesis is to be decided, because perforation, if it does take place at all, occurs generally so late, and (particularly when in the direction of the lungs) with such a slow, imperfect and harrassing evacuation of the pus by coughing, and such wasting febrile phenomena, that, instead of furnishing a cause for the delay of the operation, it increases the necessity of its performance in order to give additional and more ready drainage to the pus in the direction of the surface, and thus to shorten, and thereby to deprive, the curative process of nature of its dangerous consequences to the organism in general.

After thus establishing the very small probability of a spontaneous cure of empyema, our author arrives at the conclusion that *the accumulation of a sufficiently large quantity of pus in the pleural cavity to admit of plain recognition, furnishes a direct indication for its removal;* and that the old maxim, "Ubi pus ibi evacua," deserves full recognition in these cases.

*An early and correct diagnosis, establishing beyond a doubt the existence of a purulent acuumulation within the pleural cavity, is therefore imperatively necessary* in cases of that kind.

If the usual symptoms of œdema of the affected side of the thorax—redness of the skin in that region, the characteristic type of fever with high evening exacerbations, and more or less developed periodical rigors—are wanting, which is sometimes the case; while the physical exploration of the thorax indicates a pleuritic effusion, puncture of the thorax with an exploratory trocar, will always decide whether or not empyema exists.

In all doubtful cases the character of the accumulated fluid should thus be ascertained at once, and if pus is found, thoracentesis should be performed, as delay will always diminish the probability of a recovery.

The frequent incurability of the fundamental disease, which has caused the empyema and the high fever connected with it, should never delay, or much less set aside the operation. The effective removal of the accumulated pus will, in far the majority of instances, more than any other therapeutical measure, not only give comfort to the patient for the time he can live, but will frequently protract the fatal issue a longer or shorter period of time, if it does not accomplish a cure, and will always either lessen or check the fever with its wasting influences on the system, as the latter is almost invariably the consequence of the process of suppuration going on within a closed cavity of the organism. The operation within itself is harmless, and if death has followed it in a few instances, it is very probable that it was caused by a continuation of the morbid conditions which now and then occasion sudden death in large pleural effusions.

To prevent the admission of air when the exploring trocar is used, it is always sufficient to let the patient take a full inspiration and retain his breath the moment the trocar is withdrawn.

Our author acknowledges no valid contra-indication of thoracentesis, but such a degree of debility which would necessarily result in death without the operation in a very short time.

*The governing idea in its performance must be to have an opening amply large enough to admit free and perfect exit of the pus, and prompt and efficient cleansing of the cavity for a sufficient length of time.* The mere puncture with the trocar and canula, with exclusion of the air, does not answer the purpose. It creates too small an opening; the clearing of the cavity by syringe, even through the retained canula, is too tedious, difficult and incomplete, and this method has therefore been almost entirely abandoned, and the operation by free incision—which, although a little more difficult to perform, is certainly no more a dangerous operation than the other—is now generally preferred.

First an incision, one and a half to two inches long, through the integuments, right in the middle of an intercostal space to avoid the intercostal artery, then division of the muscles, layer by layer on the groved probe, is made. If a smaller artery bleeds, the hemorrhage is stopped by digital pressure, but the final perforation of the white, glossy pleura, which either bulges into the aperture of the incision or remains stiff and hard, is not made until all hemorrhage has been entirely arrested, as even an insignificant quantity of blood mixed with the pleural contents, gushing forth through the pleural opening, frequently causes an erroneous impression of danger. If the nature of the contents of the pleural cavity should not have been satisfactorily ascertained before, the insertion of a small trocar in one of the angles of the wound at this stage of the operation will remove any existing doubt. A free pleural incision, corresponding in size with the superficial one, finishes the operation.

The best locality for the incision, in order to avoid the obstruction of the opening by the previously depressed diaphram, is the fifth or sixth intercostal space between the mammillary and axillary lines.

Marked and immediate relief follows the operation, the respiration becomes free and easy, the fever abates, and this relief remains permanent; while, if mere puncture of the thorax has been made, the old condition of affairs, on account of imperfect drainage of the empyema, is reëstablished in a very few days, and the patient becomes clamorous for a repetition of the operation.

The after-treatment must necessarily provide for the fulfillment of the general indications of a large abscess. The cavity must be well cleansed, and the pus thereby sufficiently removed twice a day; and as decomposition of the pus—manifested by a rising of the pulse and the bodily temperature frequently beyond the degree existing before the operation—takes place within twenty-four to forty-eight hours after it in a large number of cases, the pus at the same time assuming a thin, ichorous appearance, and more or less fœtid odor. Dr. Lichtheim has made it a uniform rule to inject into the thoracic cavity twice a day, through a flexible tube, a solution of carbolic acid of one part to one hundred parts of water containing a small quantity of glycerine in order to keep the former in solution, for the purpose of arresting the secretion and decomposition of the pus. He has never observed any unpleasant symptoms under this treatment, although the urine of his patients showed by its darker color that a portion of the carbolic acid was resorbed. Subsulphate of soda, permanganate of potash and iodide of potash in solution—recommended, the first by Kussmaul, the last by Trousseau, and afterwards by Quincke—have manifestly failed in his hands to avert the decompostion of the pus, while the carbolic acid has proved very reliable for that purpose. The fluid to be injected should be warmed within the neighborhood of the usual degree of the bodily temperature before it is used. The half-sitting posture is most favorable to the performance of this procedure, and it is therefore advisable to put the patient in that position, and let him remain in it for some weeks after the operation. As the healing of the incision proceeds, the difficulties which attend thoracentesis by mere puncture from the very commencement, begin to arise and annoy the practitioner. The opening looses its lengthy form, becomes round and smaller until finally, in spite of the continued application of good large wicks from the beginning, and in spite of repeated dilatation of the opening with laminaria and sponge tents, it will not allow the fluid injected through the permanently remaining flexible catheter to run out by its side. Then it is time to insert a short double tube in place of the catheter, so that the fluid injected by one of its channels can run out again through the other.

Roser and Quincke have introduced a method and apparatus by which the pleural cavity is washed out by the introduction of air and water alternately, one driving out the other. Dr. Lichtheim describes the apparatus and its mode of operation; but as in our judgment a Davies syringe, attached to one-half of the double tube, resting in the thoracic opening, permits, in a much simpler manner, the alternate introduction of air or water into the thoracic cavity by respectively leaving the suction end out of or putting it into the fluid to be injected, it appears unnecessary to dwell on their mode of accomplishing this object.

Where the edges of the thoracic opening do not heremetically close round the double tube, this method of cleaning the thorax can, of course, not be used. Quincke substitutes in such cases a gutta percha ring pessary, in the center of which the double canula is hermetically inserted. It admits the exclusion of air by being pressed against the walls of the thorax. He has, in this manner, frequently washed out the pleural cavity immediately after the operation, and claims that it is preferable to the common mode, as it does not require the patient to turn on his side. Changes of position, immediately after the operation, are considered by him fraught with some danger, and he refers the death of one patient to them as the cause. Dr. Lichtheim prefers the common mode of washing out the cavity, through a large opening, and reserves Quincke's method for those cases where the small size of the opening makes it necessary.

The extent to which the several thoracic and neighboring organs, and particularly the lungs, are restored to their normal position, size and physiological function, the cavity diminished and the normal appearance of the thoracic wall re-established. depends upon the age of the patient and the duration of the disease before the operation was performed.

The inclination of the opening to close up manifests itself by the fact that the secretion becomes thin and scant, diminishing to a few drops of a yellow watery fluid during the whole day. On removal of the catheter under these circumstances, it closes up rapidly.

Frequently, however, the reparative process is suspended, the general condition of the patient remains favorable, but the cavity still continues to secrete pus without any prospect of a change. Dr. Lichtheim recommends, under these circumstances, the suspension of the disinfecting injections, as long as the condition of the pus does not demand them, and the use of stimulating ones in their stead, for instance Lugol's Solution, containing one per cent. of iodine, or stronger if required.

In cases where, in consequence of the duration of the empyema, the lungs have lost their faculty to expand, all these means are fruitless, and a permanent thoracic fistula remains.

The establishment of well-defined indications for the operative treatment of non-purulent pleuritic exudations, with exception of those cases where immediate danger of suffocation demands thoracentesis, is much more difficult.

A consideration of all the factors upon which the resorption of such exudations depends—to-wit: the acute or chronic character of the disease which has caused it, the condition of the blood of the patient, the character of the exuded fluid, the degree of pressure existing in the pleural cavity, etc.—teaches that it is an entire impossibility to predict, with any reasonable degree of accuracy, the final termination of a pleuritic exudate, and leaves us only in possession of one undeniable fact, to wit: *that large pleuritic exudates are generally resorbed with great difficulty, if resorbed at all, and are, therefore, often adapted to operative removal.*

Dr. Lichtheim further considers that thoracic puncture, properly practiced, is an entirely harmless operation, and states most positively that in a very large number of operations performed or witnessed by him, only in a few instances, after repeated punctures in the same individuals, the fluid became slightly turbid, and contained, microscopically examined, a few more cells; but that it has not been followed, within the scope of his personal observation, in a single instance, by acute purulent degeneration of the exudate.

He arrived at the conclusion that in robust individuals *extensive pleuritic exudations*—where resorption, although not impossible, would evidently be very tedious, and too slow to prevent the accumulation of injurious consequences to the organism—*should be removed by thoracentesis if within the first or second week after the cessation of pain and fever the exudate does not begin to diminish.* The quantity of the exudate can only be correctly estimated, not by its level alone, but by the expansion of the affected side of the thorax, the depression of the diaphragm, the displacement of the mediastinum and, in left-sided effusions, by the considerable displacement of the heart to the right. In exudates following phthisis or chronic nephritis, the operation is contra-indicated unless immediate danger of suffocation demands it.

One method for the removal of non-purulent pleuritic exudations is now generally accepted. It is that of puncture of the thorax with exclusion of the air, which will enter with each inspiration as soon as the pressure within the pleural cavity is no greater than that of the atmospheric air. This exclusion of air, although the entrance of a few air bubbles by some imperfection of the apparatus is of no consequence whatever, is essential for the prevention of suppuration within the pleural cavity.

The number of contrivances which have been invented to accomplish this object is not inconsiderable, and several of them are most probably known to every practitioner. Piorry's method is probably the most preferable one.

Dr. Lichtheim warns against the propositions of Hoppe Seiler, who, in order to accomplish complete evacuation of the serum, thought it proper to introduce a diluted solution of chloride of sodium into the thoracic cavity, by aspiration through a gutta-percha tube, to occupy the place of the remaining serum. He considers it by no means certain that such operations, to which the performance of thoracentesis in the warm water bath below the surface of the water belongs, furnish better results than the simple puncture without aspiration of fluid; but holds that if evacuation of the serum by thoracentesis is not followed by a new accumulation of fluid, the remaining serum will be resorbed, while the puncture with aspiration proposed by Hoppe Seiler, is not so absolutely harmless as the simple puncture alone, but induces, according to the history of his cases, septic decomposition of the contents of the pleural cavity (piopneumothorax).

When a large trocar is used, the escape of the serum should be frequently interrupted to prevent rupture of the fine capillaries of the newly-formed connective tissue, and a bloody color of the escaping serum should always induce the operator to exercise caution in this direction.

Dr. Lichtheim performs thoracentesis by puncture at the same locality at which he operates by incision for empyema. The simplicity of the operation excludes the possibility of serious accidents. If pus emanates from the canula after the withdrawal of the trocar, the operation by incision should be performed at once. Hemorrhagic exudations, being an evidence of tuberculosis or scirrhus of the pleura, establish an unfavorable prognosis, and where they are found to exist, the escape of the fluid should also be arrested, unless symptoms of suffocation demand its removal.

As soon as the expansion of the lungs commences an irritation of the small bronchii and vesicles, by the air entering the lungs, causes an obstinate and harassing cough. Hypodermic injections of morphia before the operation will measurably prevent this trouble. Closure of the puncture, which always heals by first intention, is the only dressing needed. Great relief and no fever, or increase of it, where it existed before, follow the operation in the largest number of cases; and, even in the most unfavorable instances, febrile phenomena hardly ever occur, while the effusion, instead of diminishing, reaches within a few days again its former extent. In these cases the patient, who has once experienced the great relief following the operation, wants it repeated; but it is well enough not to be hasty in complying with his request.

Acute purulent degeneration of the remaining serum occurs very seldom; when it does, the symptoms of empyema set in, and the treatment of that condition becomes necessary.—*(Volkman's Klinische Vorträge, No.* 43, *October*, '72.*)*

---

Dealing with Lunatics.—The *Lancet* humorously says: "In view of the cruelties recently perpetrated in lunatic asylums upon helpless inmates, the recommendation of the late Mr. Charles Buxton must to many appear to be a human one. This gentleman advised that all insane persons 'should be comfortably shot;' and, with a thoughtful concession to the privileges of the clergy, he added: 'I would have it done with the utmost decorum; perhaps by the bishop of the diocese.'"

